# Effects of Chemical Pretreatments of Wood Cellulose Nanofibrils on Protein Adsorption and Biological Outcomes

**DOI:** 10.1021/acsami.5c00391

**Published:** 2025-01-30

**Authors:** Ahmad Rashad, Miina Ojansivu, Ebrahim Afyounian, Ellinor Bævre Heggset, Kristin Syverud, Kamal Mustafa

**Affiliations:** 1 Center of Translational Oral Research (TOR), Department of Clinical Dentistry, University of Bergen, Bergen 5009, Norway; 2 Prostate Cancer Research Center, Faculty of Medicine and Health Technology, Tampere University and Tays Cancer Center, Tampere University Hospital, Tampere 33520, Finland; 3 RISE PFI, Trondheim 7034, Norway; 4 Department of Chemical Engineering, Norwegian University of Science and Technology (NTNU), Trondheim 7034, Norway

**Keywords:** tissue engineering, scaffolds, proteomics, TEMPO-mediated oxidation, carboxymethylation, cell adhesion, inflammation, foreign body response

## Abstract

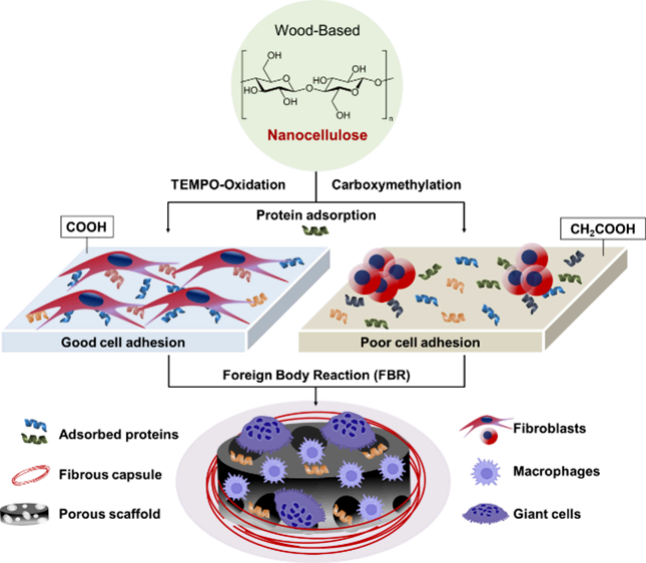

Wood-based nanocellulose is emerging as a promising nanomaterial in the field of tissue engineering due to its unique properties and versatile applications. Previously, we used TEMPO-mediated oxidation (TO) and carboxymethylation (CM) as chemical pretreatments prior to mechanical fibrillation of wood-based cellulose nanofibrils (CNFs) to produce scaffolds with different surface chemistries. The aim of the current study was to evaluate the effects of these chemical pretreatments on serum protein adsorption on 2D and 3D configurations of TO-CNF and CM-CNF and then to investigate their effects on cell adhesion, spreading, inflammatory mediator production *in vitro*, and the development of foreign body reaction (FBR) *in vivo*. Mass spectrometry analysis revealed that the surface chemistry played a key role in determining the proteomic profile and significantly influenced the behavior of periodontal ligament fibroblasts and osteoblast-like cells (Saos-2). The surface of TO-CNF 2D samples showed the highest protein adsorption followed by TO-CNF 3D samples. CM-CNF 2D samples adsorbed a higher number of proteins than their 3D counterparts. None of the CNF scaffolds showed toxicity *in vitro* or *in vivo*. However, carboxymethylation pretreatment negatively affected the adhesion, morphology, and spreading of both cell types. Although the CNF materials displayed clear differences in surface chemistry and proteomic profiles, both triggered the same foreign body response after being subcutaneously implanted in rats for 90 days. This observation highlights that the degradation rate of CNF scaffolds plays a central role in maintaining the foreign body response *in vivo.* It is imperative to comprehend the impact of chemical pretreatments of CNFs on protein adsorption and their interaction with diverse host cell types prior to the investigation of potential modifications. This knowledge is indispensable for the advancement of CNFs in regenerative applications within tissue engineering.

## Introduction

With the emergence of new biomedical fields, biomaterials have evolved from inert implants to more biologically active and responsive materials, designed for applications in cell therapy, tissue engineering, drug delivery, and biosensors.^1−3^ In tissue engineering, biomaterials are deliberately designed to serve as temporary 3D carriers (scaffolds) to deliver cells and/or biological molecules and to provide physical support for the healing process.^2^ These scaffolds are designed with specific physicochemical properties, such as functional groups, charges, wettability, topography and stiffness, that create an ideal microenvironment for tissue regeneration.^2,4^ Surface properties have been reported to significantly influence protein adsorption, cell adhesion and proliferation *in vitro.*(4,5) Before cell adhesion occurs, the initial event on the surface of biomaterials upon contact with biological fluids is protein adsorption.^6^ There is a growing body of evidence that the surface properties of biomaterials are critical determinants of the types, compositions, concentrations, and conformations of the adsorbed proteins.^6−8^ This dynamic layer of adsorbed proteins has been reported to influence subsequent biological events, including cell adhesion, proliferation, and inflammation.^9−12^

Upon *in vivo* implantation of biomaterials, immune cells immediately recognize the implanted material as a foreign body through the adsorbed proteins, thus initiating a cascade of events which may lead to a foreign body response (FBR).^13^ In many cases, this impermeable fibrous encapsulation of the implanted biomaterial can impede healing, cause tissue damage and discomfort and compromise the performance of the implant.^14,15^ However, FBR is a multifactorial, dynamic process which cannot be attributed to protein adsorption alone.^16^ Various bulk material properties, including chemical composition, size, shape, and degradation, have been shown to influence and direct FBR.^17,18^

For new types and forms of biomaterials, investigating biomaterial-cell interactions through the mediation of the adsorbed proteins and linking that to cell response *in vitro* and the host response *in vivo* is crucial for their applications. In this context, wood-based cellulose nanofibrils (CNFs) a new form of cellulosic biomaterials, are of interest.^19^ These nanocelluloses exhibit exceptional physicochemical properties that distinguish them from traditional cellulose, including unique geometrical dimensions, morphology, high specific surface area, crystallinity, orientation, and impressive mechanical properties. Because of their large aspect ratio and fiber entanglement, CNFs can easily form hydrogels, which are advantageous for the preparation of tissue engineering scaffolds.^20,21^ Among the various potential applications, it is evident that CNFs biomaterials have the capacity to function as scaffolds and membranes for periodontal bone regeneration. Nanocellulose-based membranes have demonstrated significant potential for periodontal tissue regeneration by promoting the proliferation of periodontal ligament stem cells and enhancing their osteogenic differentiation.^22^ In a recent study, Korkeamäki et al. developed a series of CNF-based porous scaffolds for the purpose of bone tissue engineering. This study demonstrated the ability of these scaffolds to support the proliferation and osteogenic differentiation of osteoblast-like cells (Saos-2).^23^

For several years, our research group, like many others, has focused on exploring the applications of nanocellulose in tissue engineering. We have applied different chemical pretreatments, including TEMPO-mediated oxidation (TO-CNF) and carboxymethylation (CM-CNF), to develop wood-based CNFs with various surface chemistries, available in multiple forms such as films, sponges, hydrogels, and bioinks for bioprinting.^20,24−27^ We investigated several biological interactions between these different scaffolds and mouse fibroblasts and human macrophage-like cells.^20,27^ Unlike TEMPO-mediated oxidation, carboxymethylation adversely influenced the morphology of these cells and limited their spreading. It is implausible that surface properties of CNFs directly influence cells responses. Instead, surface properties likely affect the composition, conformation, and concentration of proteins adsorbed on the surface, and cells respond to these changes in the adsorbed proteins.^24,28,29^

We hypothesized that the unique surface characteristics resulting from different chemical pretreatments of CNF-based scaffolds primarily influence cellular responses by modulating protein adsorption. While there are some studies on the interactions of nanocellulose-based biomaterials with various protein models, the interactions of complex protein models, such as serum, with wood-based nanocellulose of different chemistries and their impact on the biological responses of various cell types remain underexplored in the literature.^28^ Most studies focused on nanocellulose modified with other polymers, nanoparticles, or bioceramics, using simple protein models such as bovine hemoglobin and bovine serum albumin.^30−32^ Only very few reports investigated the effect pure CNFs with different carboxyl groups and carboxymethyl groups on cell response.^28,29,33,34^ However, these studies did not show intensive protein analysis and did not link protein signature to the inflammatory response of the CNFs *in vitro* and *in vivo*.

To test our hypothesis, we fabricated TO-CNF and CM-CNF, in the form of 2D surfaces and 3D porous scaffolds. These materials were then incubated in growth culture medium with fetal bovine serum (FBS) and subjected to mass spectrometry analysis to identify and quantify the adsorbed proteins. Subsequently, the correlation between protein adsorption and cellular responses was assessed by culturing different human cell types (Saos-2: osteoblast-like cells, and human periodontal ligament fibroblasts: PDLF) on the materials and evaluating their adhesion, viability, morphology, and inflammatory responses. Finally, the development of the foreign body response to the 3D porous scaffolds was evaluated in rats, 3 months after subcutaneous implantation.

## Materials and Methods

### Materials

All chemicals used in the preparation of CNFs hydrogels were procured from Sigma-Aldrich. The nanocellulose was extracted from a fully bleached softwood kraft pulp sourced from Södra, Sweden, consisting of a blend of 25% Pine and 75% Spruce. Dulbecco’s Modified Eagle Medium (DMEM) from Thermo Fisher Scientific was used to culture periodontal ligament fibroblast (PDLF) cells. The PDLF used in this study were obtained from the biobank of the Department of Clinical Dentistry, University of Bergen, Norway. Initially, these cells were isolated from tissue explants obtained during oral surgery, as previously reported in our research.^35^ Saos-2 cells and McCoy’s cell culture medium were purchased from ATCC (Manassas, VA, USA). Dulbecco’s phosphate-buffered saline (DPBS), 4′,6-diamidino-2-phenylindole (DAPI), PicoGreen kit, live/dead kit and goat antimouse Alexa Fluor 568 IgG secondary antibody were purchased from Thermo Fisher Scientific. Lactate dehydrogenase (LDH) toxicology kit and Fluo-8 calcium flux kit were purchased from Abcam. Mouse monoclonal anti-β-actin, HRP-conjugated goat antimouse IgG, and goat antirabbit IgG secondary antibodies were purchased from Santa Cruz Biotechnology. Recombinant rabbit vinculin antibody was purchased from Life Technologies.

### Nanocellulose Preparation and Characterization

#### Preparation of the Hydrogels

Nanocellulose hydrogels were prepared by mechanical fibrillation after chemical treatment of softwood pulp according to our previously described method.^20^ Briefly, the oxidation reaction was catalyzed using 2,2,6,6-tetramethylpiperidinyl-1-oxyl (TEMPO) in conjunction with sodium hypochlorite. For carboxymethylation, the pulp was soaked in a mixture of monochloroacetic acid and isopropanol. Sodium bicarbonate was then added to convert the carboxyl groups to the sodium form. After both chemical treatments, the pulp was homogenized at a pressure of 1000 bar using a Rannie homogenizer (SPX Flow Technology, Silkeborg, Denmark). Finally, the density of the surface groups (aldehyde, carboxyl and carboxymethyl) was determined by conductometric titration.^20^

#### 2D Surfaces and 3D Scaffolds Preparation and Characterization

To prepare 2D surfaces, tissue culture plates (TCP; Nunc, Thermo Fisher Scientific) were coated with CNFs suspensions (0.33% solid content) and incubated at 37 °C for 12 h to evaporate the water. The surfaces were analyzed by light microscopy (Nikon Eclipse Ti, Tokyo, Japan). For atomic force microscopy (AFM; diMultiMode V, Bruker, U.S.A.), nanocellulose suspensions (0.01%) of each sample were added to clean mica surfaces and allowed to dry in air.^36^ The average surface roughness (Ra) was obtained from the images (10 μm × 10 μm) using NanoScope Analysis software (version 1.9).

To evaluate the wettability of the CNFs surfaces, their water contact angles were measured using a Contact Angle and Surface Tension Meter (KSV Instruments, Finland) with a 3 μL drop of water.^26^ The surface charges of nanocelluloses in cell culture media were measured using a Zetasizer (Malvern Instruments, Malvern, UK). To prepare 3D porous scaffolds, CNFs suspensions were poured into 48-well plates and frozen at −20 °C for 24 h and then lyophilized for 24 h. The structure of the scaffolds was examined by scanning electron microscopy (SEM, JEOL JSM-7400F, Japan) at 5 kV after sputter coating with platinum.^27^ To determine the compressive modulus, cylindrical cast 3D porous nanocellulose scaffolds were compressed at 0.1 mm/s using a texture analyzer (TA.XT.-Plus Texture Analyzer, Stable Micro Systems, Surrey, UK).^36^

#### Proteomic Analysis of Adsorbed Serum Proteins

To examine protein adsorption on biomaterial surfaces, both 2D and 3D samples were immersed in DMEM supplemented with 10% fetal bovine serum (HyClone, GE Healthcare, Utah, U.S.A.) and incubated at 37 °C for 24 h. Tissue culture plates were used as controls. After incubation, the samples were washed with DPBS and then incubated in 2% sodium dodecyl sulfate (Sigma-Aldrich) for 20 h. Total protein content was quantified using a commercially available protein assay kit (Pierce BCA Protein Assay, Rockford, IL, U.S.A.) according to the manufacturer’s instructions. Label-free mass spectrometry was then used to determine the relative abundance of proteins adsorbed on the nanocellulose surfaces, as previously described.^37^ Protein solutions were lyophilized and mixed with urea solution for unfolding. After trypsin digestion, the samples were purified, lyophilized and subjected to mass spectrometric analysis using a Dionex Ultimate NCS-3500RS liquid chromatography system (Sunnyvale, CA, USA). After protein identification, filtering, and mapping, the raw data were processed using Perseus (version 2.0.9.0). We performed a log2 transformation without additional normalization, as the relative label-free quantification (LFQ) intensity column already includes normalized abundance values. The processed data underwent differential expression analysis (DE) using ANOVA. A list of differentially expressed proteins was generated and subjected to Post hoc Tukey’s Honestly Significant Difference (HSD) test, with a False Discovery Rate threshold of 0.05. These proteins were then grouped into clusters based on the Euclidean distance metric and Average Linkage method. The data table was exported to a .txt file for further analysis in Python, enabling additional analyses beyond those available in Perseus. Python was used to identify the unique and overlapping adsorbed proteins in all groups.

For each protein, it was considered adsorbed in each treatment if at least one biological replicate had a value greater than 0. This data was then used to generate Venn diagrams and heatmaps.

### Cell-Nanocellulose Interactions

#### Cell Culture

Human periodontal ligament fibroblast (PDLF) cells at passages 5–8 were grown in DMEM supplemented with 10% FBS. Saos-2 cells at passages 9–12 were grown in McCoy’s medium supplemented with 15% FBS. Both growth media were supplemented with antibiotics (100 U/ml penicillin and 0.1 mg/ml streptomycin) and the cells were maintained at 37 °C in a 5% CO_2_ atmosphere.

#### Cell Adhesion, Viability and Morphology

Saos-2 or PDLF cells were seeded for 3 days on either 2D CNFs surfaces (coated 24-well plates) or 3D scaffolds (10 mm × 3 mm). Tissue culture polystyrene (TCP) plates were used as controls. To assess cell adhesion, cells were harvested after 24 h in 0.1% Triton-X/PBS solution and stored at – 80 °C. Cell numbers were quantified using a dsDNA assay (PicoGreen) according to the manufacturer’s protocol. Fluorescence was measured at 480/520 nm using a microplate reader (FLUOstar OPTIMA, BMG LABTECH, Germany).^20^ To evaluate the toxicity, lactate dehydrogenase (LDH) release was measured from the culture medium at days 1 and 3, using a colorimetric kit (Abcam, Cambridge, UK).^26^ On day 3, cell viability was analyzed by live/dead staining. Samples were incubated in a working solution of EthD-1 (stains dead cells red) and Calcein-AM (stains living cells green) for 45 min and then imaged with a fluorescence microscope (Nikon Eclipse Ti, Tokyo, Japan).^26^ To assess cell morphology on 2D surfaces on day 1, cells were fixed with 4% paraformaldehyde and stained with phalloidin-Atto488 (dilution 1:50) and DAPI (dilution 1:2000) for 45 min at room temperature (RT). To further investigate the influence of CNFs on cell morphology, only half of each well was coated with CNFs materials and the cells were seeded for 2 days before fixation and staining with phalloidin-Atto488. Cell morphology on the 3D scaffolds after 3 days was visualized by scanning electron microscopy. Samples were fixed in 3% glutaraldehyde, vacuum-dried, sputter-coated with platinum and imaged at 5 kV.

#### Immunostaining

Immunocytochemical staining was used to monitor the production and distribution of vinculin after 1 and 3 days of culture. After fixation with 4% paraformaldehyde and permeabilization with 0.1% Triton X-100, samples were blocked with 1% bovine serum albumin, then treated with mouse monoclonal antivinculin (1:400 dilution) and incubated for 2 h at room temperature (RT) with shaking. Simultaneously, the actin cytoskeleton was stained with phalloidin-Atto488 (dilution 1:50) and secondary antibody (goat antimouse Alexa Fluor 568 IgG; dilution 1:250) was applied for 45 min at RT. Cell nuclei were counterstained with DAPI (1:2000 dilution). Finally, the samples were visualized using a Nikon inverted fluorescence microscope.

#### Western Blot

Production of actin and vinculin was assessed by Western blot analysis after 3 days of culture. Samples were lysed using Laemmli buffer (Bio-Rad, CA, U.S.A.) and proteins were separated by sodium dodecyl sulfate-polyacrylamide gel electrophoresis. The proteins were transferred to a polyvinylidene fluoride membrane (Healthcare, U.K.) and blocked with a nonfat milk solution. The membrane was then probed with mouse monoclonal anti-β-actin (1:2000 dilution; loading control) and recombinant rabbit vinculin ABfinity antibody (1:2000 dilution). Goat antimouse IgG and goat antirabbit IgG HRP-conjugated secondary antibodies (1:2000 dilution) were used. The target proteins were visualized using a chemiluminescence reagent (ECL; GE Healthcare) and imaged using the ChemiDoc MP imaging system (Bio-Rad, CA, USA).

#### Determination of Intracellular Calcium Levels

A Fluo-8 Calcium Flux Assay Kit (Abcam), containing a membrane-permeable calcium indicator, was used in accordance with the manufacturer’s instructions. Cells were cultured on both tissue culture polystyrene (TCP) and CNF-coated TCP in 96-well plates for 4 h. They were then treated with Fluo-8 working solution for 30 min in a cell incubator, followed by an additional 30 min of incubation at room temperature, in the dark. The cells were then examined using a fluorescence microscope and the fluorescence intensity was quantified using a microplate reader at 490/525 nm.

#### Production of Inflammatory Mediators

Cells (5 × 10^5^) were seeded on scaffolds (20 mm × 4 mm) in 6-well plates and cells cultured on tissue culture plates served as controls. The levels of inflammatory mediators in the supernatant were measured after 1 and 3 days, using a Bio-Plex human kit (Bio-Rad Inc., Hercules, CA, USA) and a multiplex system (Bio-Plex200, Bio-Rad), according to the manufacturer’s instructions and our previously published report.^27^

#### Subcutaneous Implantation in Rats

The animal experiment was approved by the Norwegian Animal Research Authority (FOTS Reference No: 8035). Scaffolds (10 mm × 4 mm) were implanted subcutaneously in the dorsal area of 6 female Wistar rats, as previously described.^27^ After 90 days, samples were harvested and fixed with 4% paraformaldehyde. Finally, samples were embedded in paraffin, sectioned, and stained with hematoxylin and eosin (H&E).

#### Statistical Analysis

Statistical analysis was undertaken using independent samples *t* test or one-way ANOVA with Tukey’s post hoc comparison of means, using GraphPad Prism software. Data are expressed as mean ± standard deviation (SD). Differences were considered statistically significant at *p* ≤ 0.05.

## Results

### Characterization of CNFs Materials

**Figure 1:**
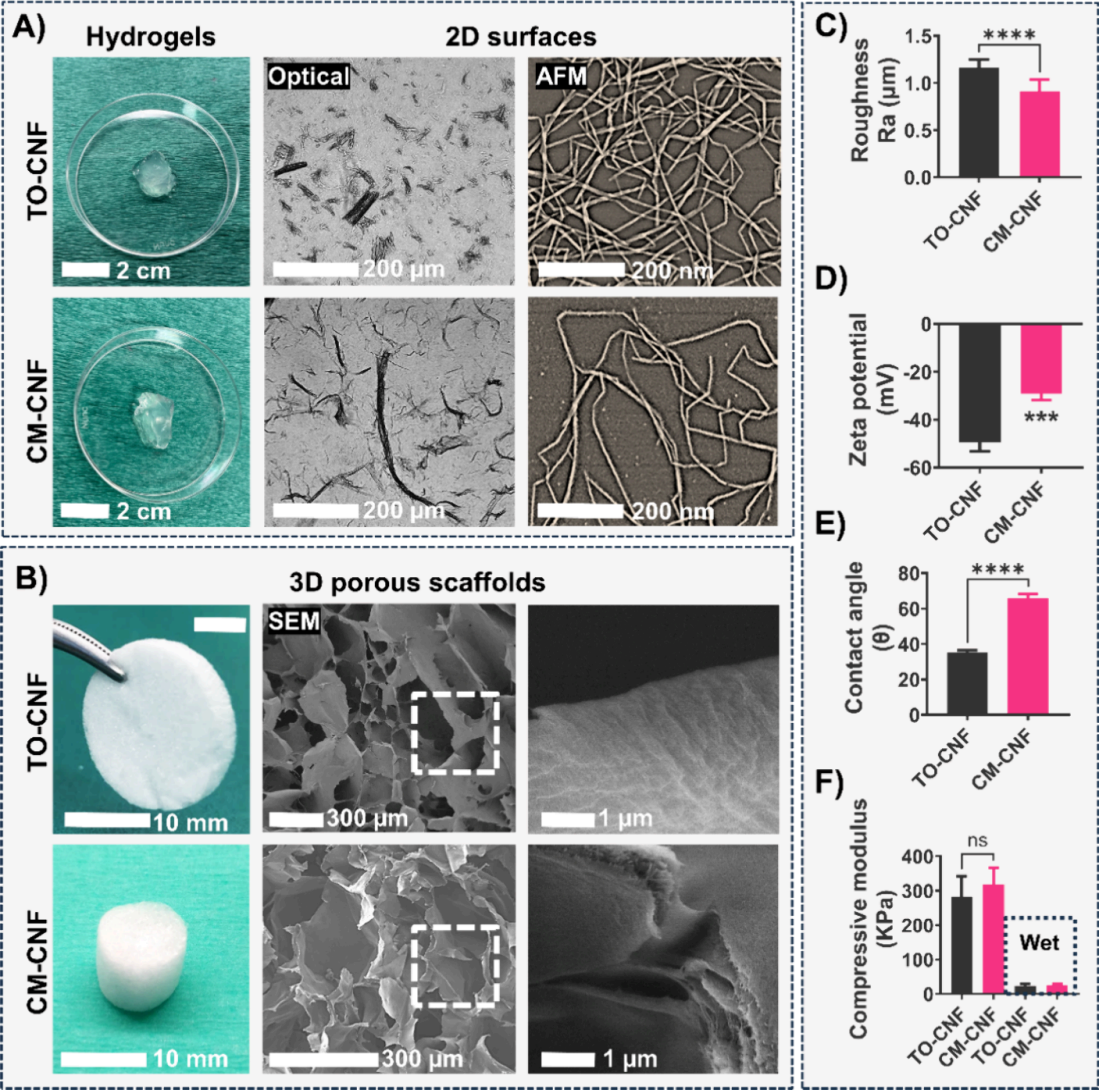
Bulk and surface characterization of the prepared CNFs materials. (A) Hydrogels (as prepared), microfiber analysis by optical microscope, nanofiber analysis by atomic force microscopy (AFM). (B) 3D porous scaffolds after freeze-drying and scanning electron microscopy (SEM) evaluation of the pores and the surface of the scaffolds (C) Roughness average (Ra) (*n* = 5). (D) Zeta potential (*n* = 3). (E) Water contact angle (*n* = 5). (F) Compressive elastic modulus of wet and dry scaffolds (*n* = 5). The graphs illustrate mean ± SD. Significance levels were p < 0.001 (****), and p < 0.005 (***).

Using TEMPO-mediated oxidation and carboxymethylation pretreatments, hydrogels of cellulose nanofibrils were prepared, with a solids content of 1% and a hierarchical fibrous structure, along with different surface functionalities (Figure 1).

As previously documented, TO-CNF contained aldehyde groups (CHO = 211 ± 60 μmol/g), while CM-CNF contained carboxymethyl groups (CH_2_COOH = 346 ± 26 μmol/g). TO-CNF had a higher concentration of carboxyl groups (COOH = 764 ± 60 μmol/g) than CM-CNF (58 ± 1 μmol/g).^20^ Various microscopy techniques revealed that the prepared cellulose nanofibrils exhibited a multiscale fibrous structure, ranging from micrometers to nanometers (Figure 1A and B). Optical micrographs showed substantial fiber fragments with surface fibrillation, while maintaining their overall size integrity. CM-CNF samples showed a greater abundance of these fragments than TO-CNF samples. AFM imaging confirmed the nanometer diameter of the fibrils. When configured into 3D scaffolds, both CNFs samples exhibited internal porous architectures with pores larger than 100 μm. Previously, these scaffolds were found to have pore sizes ranging from 7 to 590 μm.^27^

SEM analysis allowed closer examination of the scaffolds, revealing submicron fibrillar surfaces. Overall, the structural characterization confirmed the hierarchical nature of the prepared CNFs samples. Figure 1C illustrates the submicron roughness of the samples after quantification. TO-CNF showed a slightly higher roughness (Ra = 1016 ± 80 nm) than CM-CNF (Ra = 900 ± 120 nm). In addition, TO-CNF had a significantly more negative surface charge (−49.3 ± 4.7 mV) than CM-CNF (−29 ± 3.6 mV) (Figure 1D). In terms of wettability, the surface of CM-CNF was less hydrophilic (65° ± 1.1) than TO-CNF (35.3° ± 0.4), as shown in Figure 1E. In addition to surface characterization, the bulk mechanical properties of the 3D scaffolds were evaluated under wet and dry conditions (Figure 1F). In the dry state, the mean compressive modulus of the TO-CNF scaffold was slightly lower (282 ± 60 KPa) than that of the CM-CNF (319 ± 48 KPa). However, these values decreased significantly on water absorption and were nearly equivalent (24 ± 6 KPa and 25 ± 5 KPa, respectively).

### Proteomics Analysis

**Figure 2:**
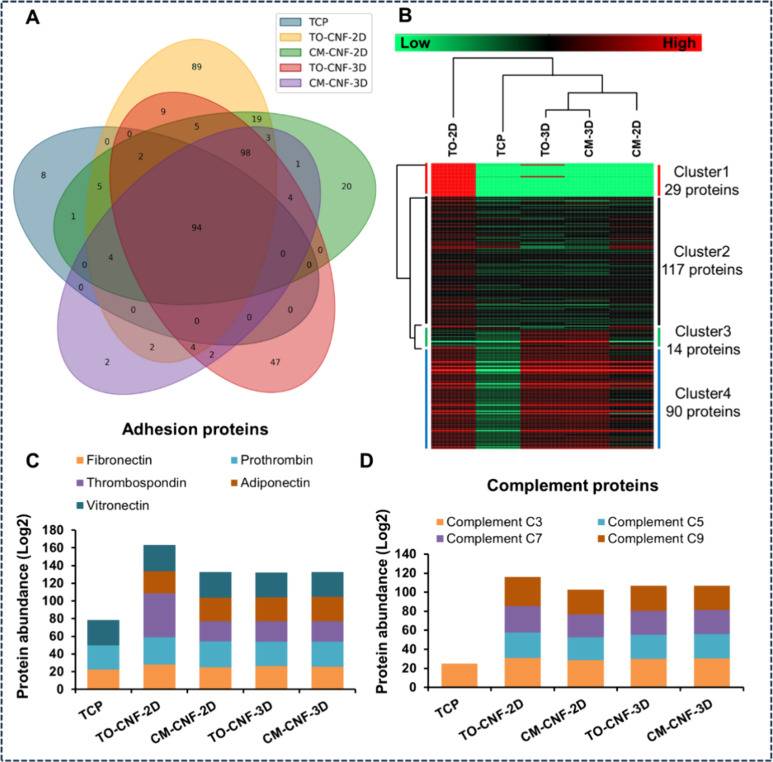
Proteomic analysis of the protein adsorbed on the surface of CNFs biomaterials and tissue culture plates (TCP). (A) Venn diagram of the serum proteins and the overlaps identified on the different CNFs biomaterials. (B) Hierarchical clustering of differentially expressed proteins visualized as a heat map of 250 proteins (ANOVA, *p < 0.05*). High and low expressions are shown in red and green respectively. (C) Abundance of adhesion and cytoskeleton regulating proteins. (D) Abundance of adsorbed complement system proteins.

Protein adsorption was greatly influenced by both the shape and chemistry of the samples (Figure 2). TCP showed the lowest number of proteins (114), while the surface of TO-CNF 2D samples showed the highest protein adsorption (334), followed by TO-CNF 3D samples (265). CM-CNF 2D samples adsorbed a greater number of proteins (256) than their 3D counterparts (214). A Venn diagram depicting the adsorbed proteins (Figure 2A) revealed 94 proteins common to all groups. Moreover, the number of uniquely adsorbed proteins was 8 for TCP, 89 for TO-CNF-2D, 20 for CM-CNF-2D, 47 for TO-CNF-3D and 2 for CM-CNF-3D.

A comparative analysis of protein expression was undertaken to assess intergroup differences in protein abundance (Table 1). The most pronounced differences were observed between the TO-CNF and TCP groups, with 160 significant proteins showing differential expression. In terms of surface chemistry, there were 68 significant differences in proteins adsorbed on TO-CNF-2D compared to CM-CNF-2D. However, this number decreased to 4 for the 3D configurations of the scaffolds. We then categorized these differentially expressed proteins into four distinct clusters, as shown in the heat map in Figure 2B. Figures 2C and 2D show the influence of sample chemistry and shape on the relative abundance of specific proteins (prothrombin, fibronectin, vitronectin, thrombospondin, adiponectin, fibrinogen and complement) known to be involved in various adhesion and inflammatory pathways. Overall, TO-CNF-2D samples showed higher levels of these proteins than the other groups and TCP surfaces showed the least protein adsorption.

**Table 1:** Differential Protein Expression Analysis of Adsorbed Proteins

	TCP	TO-CNF-2D	TO-CNF-3D	CM-CNF-2D	CM-CNF-3D
TO-CNF-2D	160		86	68	86
TO-CNF-3D	114	86		18	4
CM-CNF-2D	44	68	18		24
CM-CNF-3D	109	86	4	24	

### Cell Adhesion, Viability and Morphology

**Figure 3:**
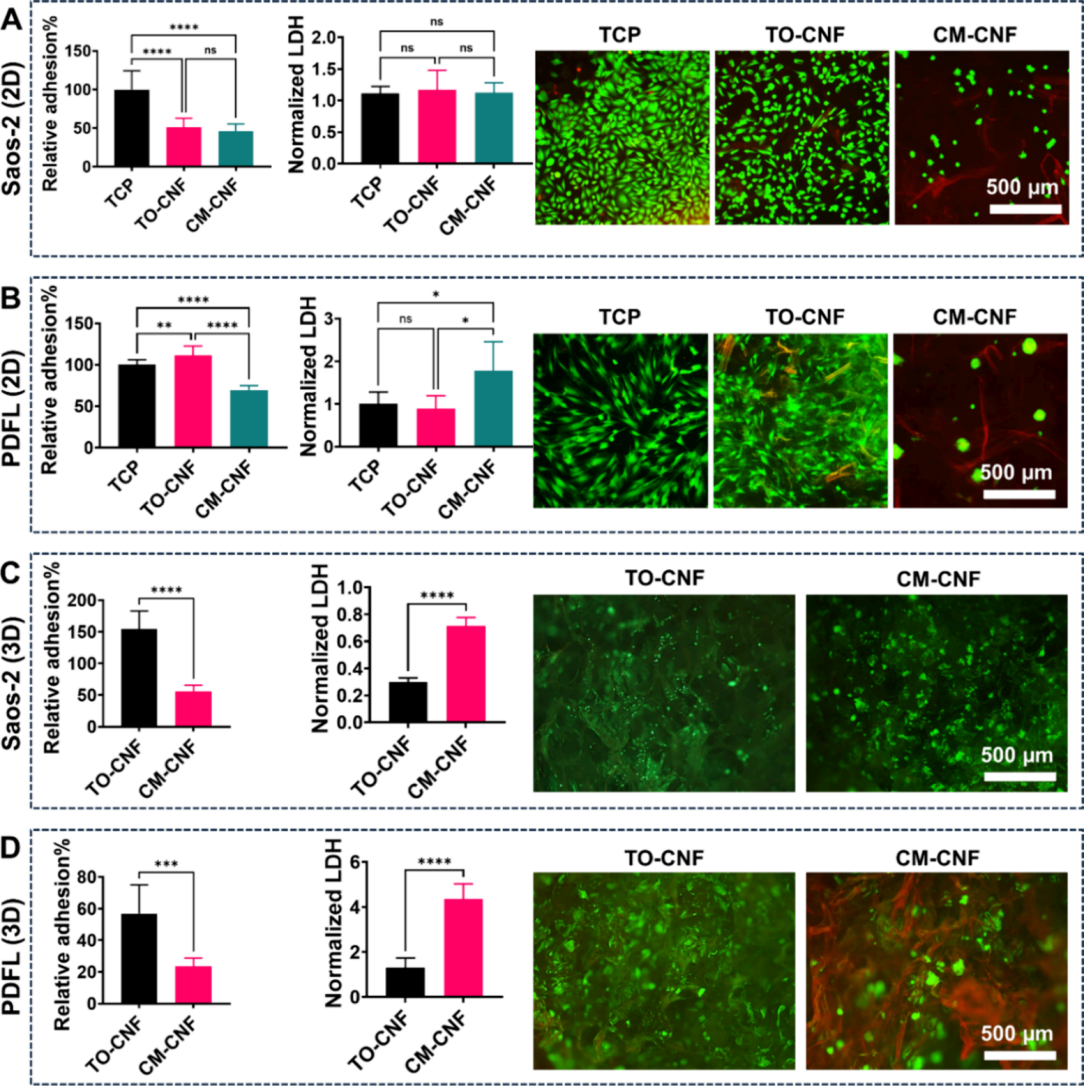
Cell adhesion by PicoGreen assay after 1 day, cell cytotoxicity by LDH assay after 1 day, and cell viability by live/dead staining after 3 days of culture on 2D and 3D CNFs samples. (A) Saos-2 cultured on CNFs 2D surfaces relative to the TCP (*n* = 5). (B) PDLF cultured on CNFs 2D surfaces relative to the TCP (*n* = 5). (C) Saos-2 cultured on CNFs 3D scaffolds (*n* = 5). (D) PDLF cultured on CNFs 3D scaffolds (*n* = 5). All values are expressed as mean ± SD. Significance levels were *p < 0.001* (****), p < 0.005 (***), p < 0.01 (**) and p < 0.05 (*).

After 1 day of incubation, significantly fewer Saos-2 cells adhered to the CNF-2D surfaces than to the TCP surfaces, regardless of their chemical composition (Figure 3A). Conversely, significantly more fibroblasts adhered to the TO-CNF surfaces than to the other groups (Figure 3B), indicating different cellular responses to the same biomaterial. Regarding cytotoxicity, there were no significant intergroup differences in LDH release from Saos-2 cells (Figure 3A). However, the release from fibroblasts cultured on CM-CNF 2D surfaces was significantly higher than that from other groups. Furthermore, cytocompatibility was confirmed by live/dead staining, which showed excellent viability of both cell types on all samples, with almost all cells staining green (indicating live cells) (Figure 3).

On the 3D scaffolds, both cell adhesion and LDH release were significantly different on TO-CNF than on CM-CNF (Figure 3C and 3D). Both cell types showed robust viability, with most cells staining green. However, live/dead staining revealed a distinct cell morphology on all CM-CNF samples, with clusters of round cells, in contrast to TCP and TO-CNF samples.

**Figure 4:**
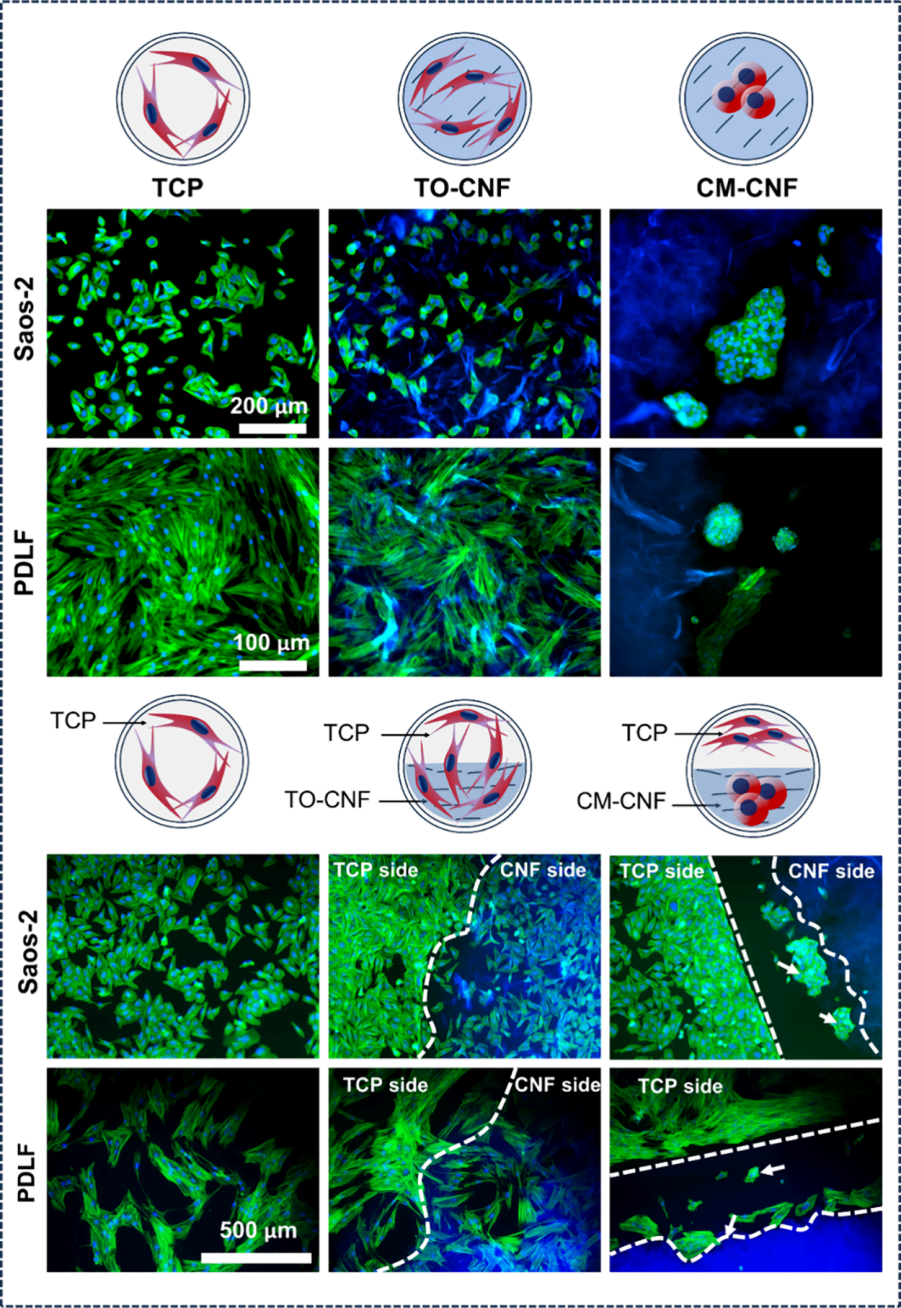
Fluorescence microscopy images taken on day 2 show F-actins (green) of Saos-2 and PDLF cells cultured on CNFs and TCP surfaces. DAPI was used to stain nuclei, while nanocellulose was stained blue. White arrows indicate cell clusters.

To confirm these morphological observations, phalloidin staining was used to examine the organization of the cytoskeleton in both cell types. As shown in Figure 4, cells tended to avoid the CM-CNF surface, forming clusters at the border of the CM-CNF area, followed by a zone free of cells. On the TCP surface, most cells showed elongation and robust spreading. Apparently, CM-CNF inhibited cell adhesion and spreading more than TO-CNF and TCP. Cells on TCP and TO-CNF surfaces exhibited a well-organized cytoskeleton characterized by aligned and straight actin fibers.

**Figure 5:**
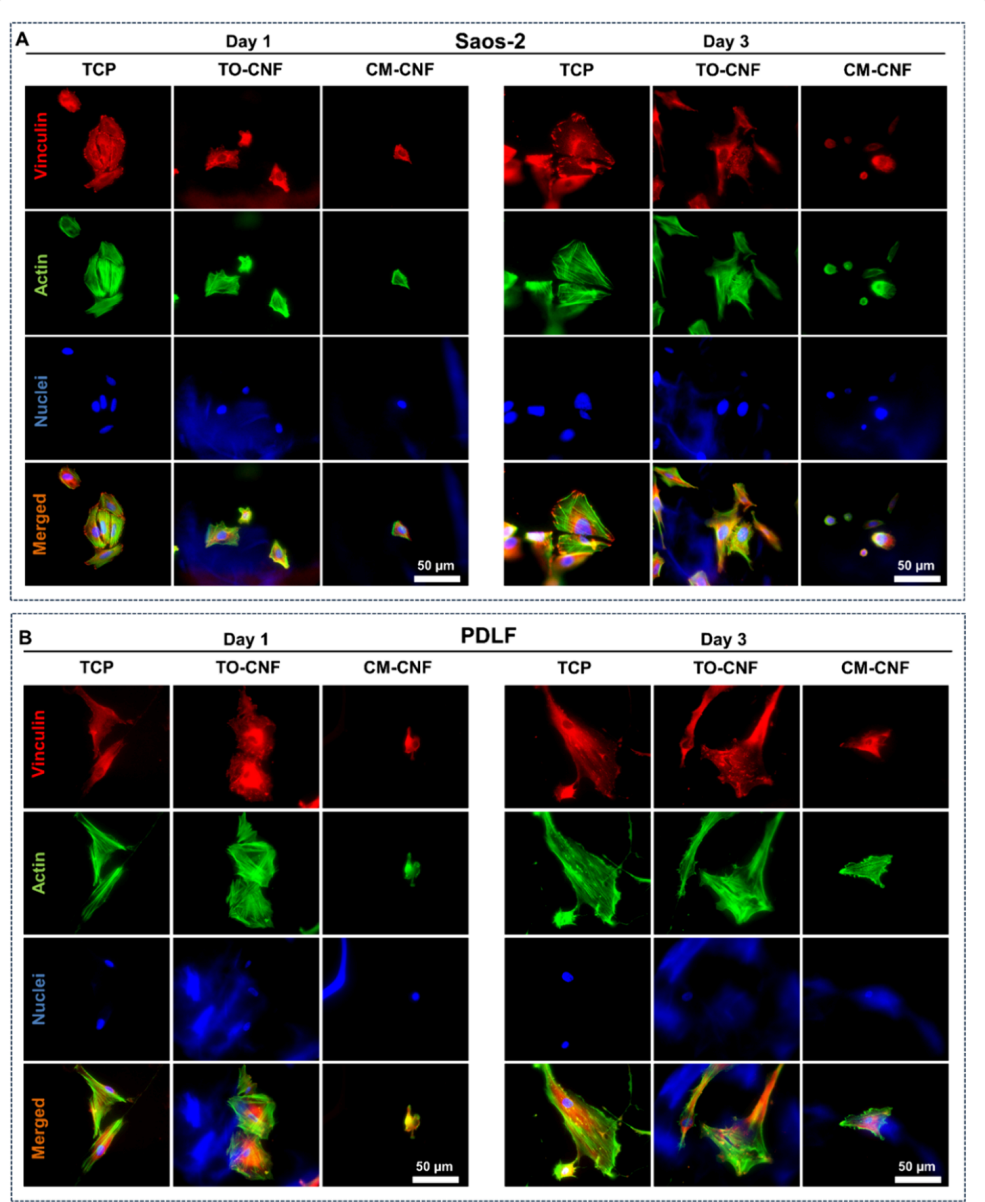
Morphology of Saos-2 cells (A) and PDLF (B) on TCP and 2D CNFs surfaces was examined after 1 and 3 days of culture, revealing the organization of the cytoskeleton f-actin (green) and the distribution of vinculin (red). DAPI was used to stain the nuclei, while nanocellulose was stained blue.

**Figure 6:**
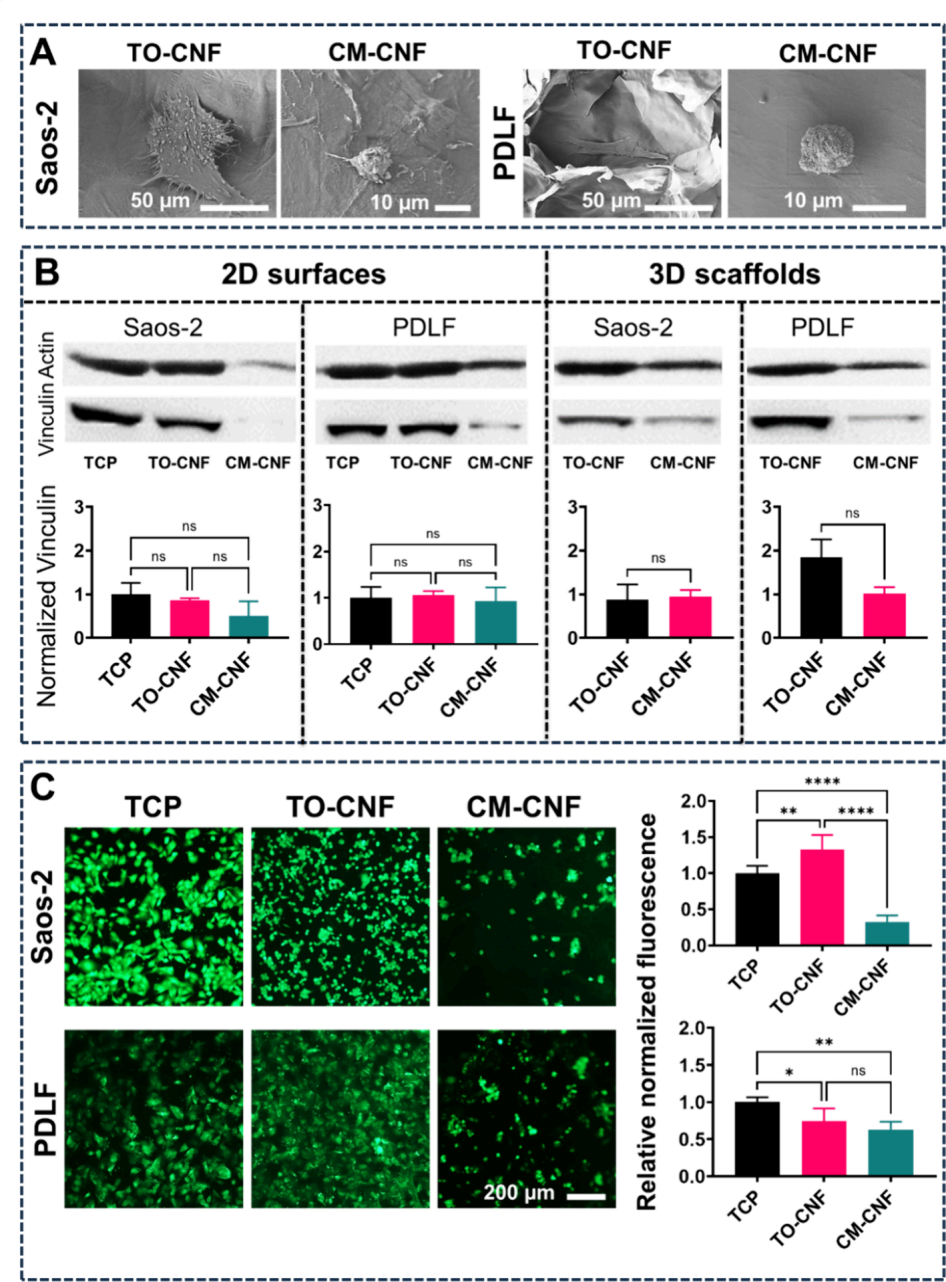
Cell morphology by SEM (A) and cytoskeletal protein production by Western blot after 3 days of culture (B). Normalized vinculin production by Western blot (*n* = 2). (C) Normalized intracellular calcium levels (*n* = 4). All data are expressed as mean ± SD. Significance levels were p < 0.001 (****), p < 0.01 (**) and p < 0.05 (*).

Immunostaining (Figure 5) and Western blot (Figure 6) techniques were then applied to assess the production and distribution of vinculin by the adherent cells. Vinculin, a focal adhesion protein, binds to actin and plays a fundamental role in integrin-mediated cell adhesion. Immunofluorescence images revealed smaller cells with fewer focal adhesion sites on CM-CNF than on TCP and TO-CNF surfaces on both days 1 and 3 (Figure 5). SEM analysis confirmed the small, round shape of both cell types on CM-CNF 3D porous scaffolds. In contrast, cells on TO-CNF scaffolds showed well-developed filopodia that adhered to the surrounding environment (Figure 6A). Western blot results indicated that Saos-2 cells produced more vinculin on TCP and TO-CNF 2D surfaces (Figure 6B), while on the 3D scaffolds, vinculin production was similar. In the case of fibroblasts, the production of vinculin by PDLF cultured on CM-CNF surfaces was comparable to that on other 2D surfaces, but significantly lower on the 3D scaffolds.

There is now considerable evidence that both integrin-mediated and nonintegrin-induced calcium signaling are critical for the rapid and sustained modulation of cell-cell and cell-substrate interactions during adhesion, spreading, and migration of various cell types.^38^ With respect to intracellular calcium concentrations, calcium ions were detected in both cell types, cultured on all surfaces. After adjustment for cell numbers, intracellular calcium levels were lower in both cell types in the CM-CNF group than in the other groups. Saos-2 cells cultured on TO-CNF had significantly higher intracellular calcium levels than those on TCP and CM-CNF surfaces. Conversely, PDL fibroblasts cultured on TCP surfaces had significantly higher calcium levels than those on other surfaces (Figure 6C).

### *InVitro* Inflammatory Response

**Figure 7:**
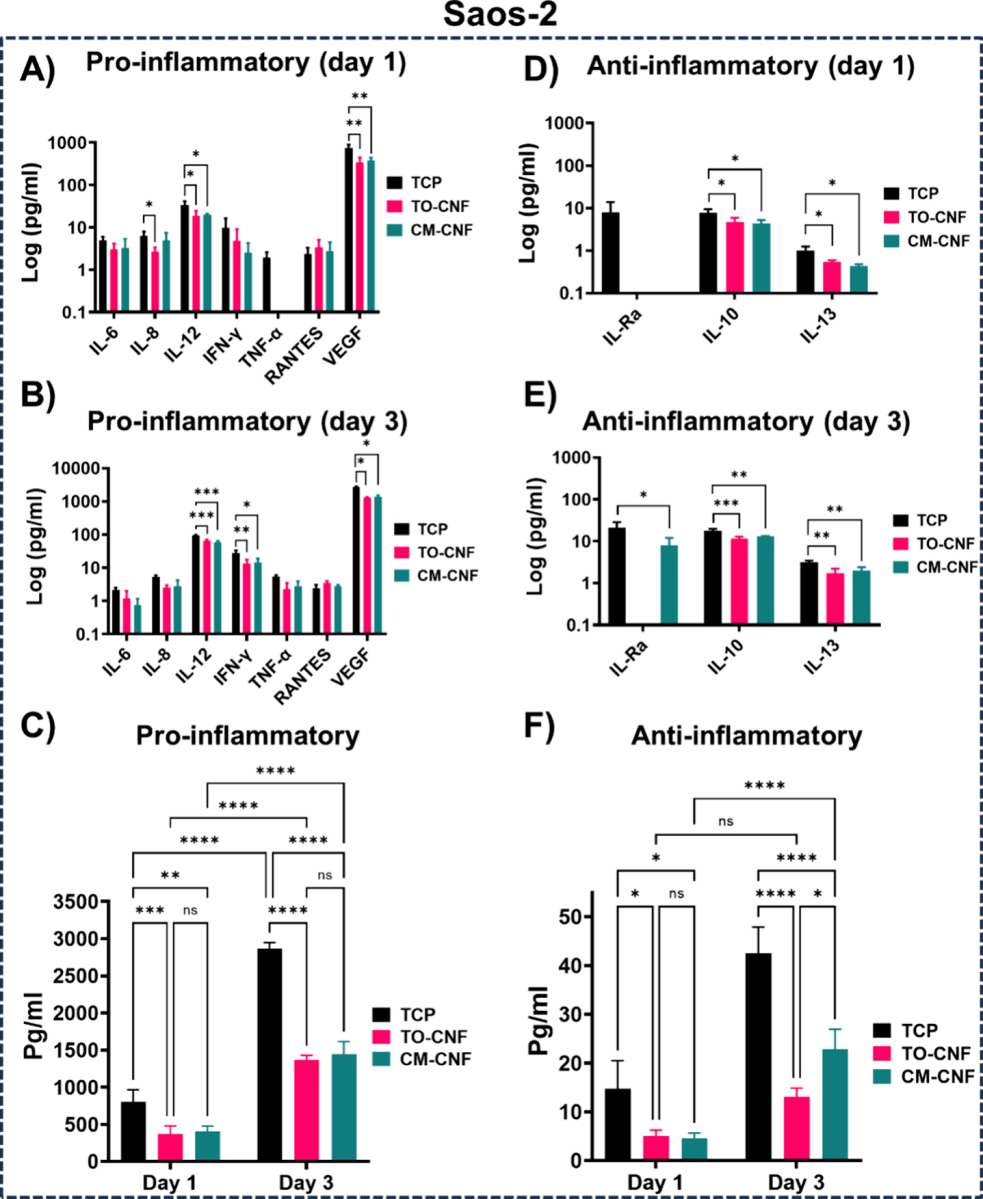
Production of inflammatory mediators from Saos-2 cells. (A) Proinflammatory markers on day 1 (B) Proinflammatory markers on day 3. (C) Grouped proinflammatory markers on days 1 and 3. (D) Anti-inflammatory markers on day 1 (E) Anti-inflammatory markers on day 3. (F) Grouped anti-inflammatory markers on day 1 and 3 (*n* = 4). All values are expressed as mean ± SD. Significance levels were p < 0.005 (***), p < 0.01 (**) and p < 0.05 (*). IL: Interleukin, ILR: Interleukin receptor, IFN: Interferon gamma, TNF: Tumor necrosis factor, RANTES: Regulated upon activation, normal T-cell expressed and presumably secreted. VEGF: Vascular endothelial growth factor.

**Figure 8:**
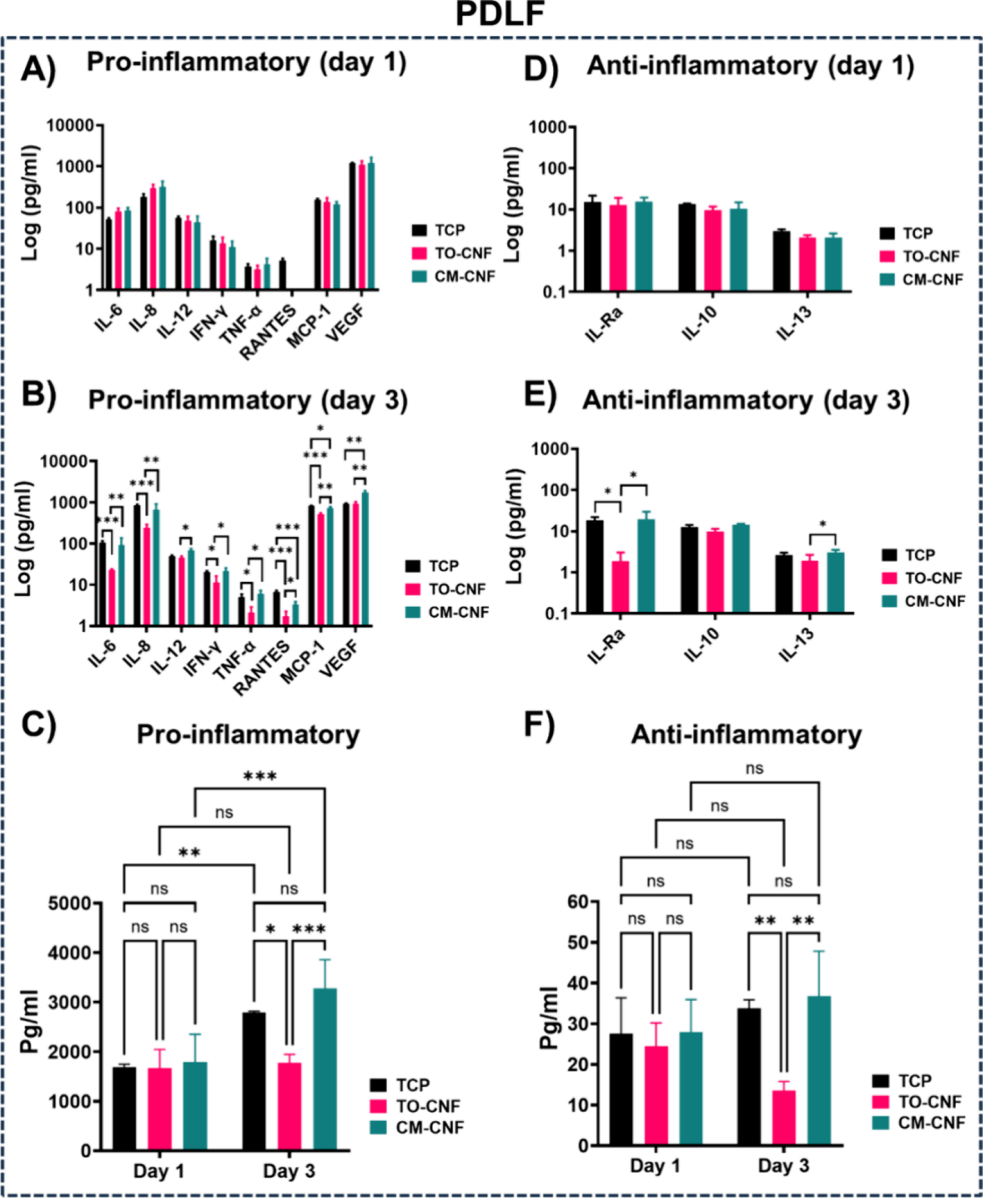
Production of inflammatory mediators from PDLF cells. (A) Proinflammatory markers on day 1 (B) and day 3. (C) Grouped proinflammatory markers on days 1 and 3. (D) Anti-inflammatory markers on day 1 (E) Anti-inflammatory markers on day 3. (F) Grouped anti-inflammatory markers on day 1 and day 3 (*n* = 4). All values are expressed as mean ± SD. Significance levels were p < 0.005 (***), p < 0.01 (**) and p < 0.05 (*). MCP: Monocyte chemoattractant protein.

The production of proinflammatory and anti-inflammatory mediators is presented in Figures 7 and 8. There were no significant differences between TO-CNF and CM-CNF in the production of pro- and anti-inflammatory mediators by Saos-2 cells (Figure 7 A and B). Compared to TCP on both day 1 and day 3, one or both CNFs scaffolds significantly decreased the production of pro-inflammatory mediators (IL-8, IL-12, and VEGF). In addition, over time IL-6 levels decreased while VEGF and IL-10 levels increased significantly, regardless of surface chemistry. In terms of anti-inflammatory mediators (IL-Ra, IL-10, and IL-13), TCP prompted Saos-2 cells to produce a greater number of markers than CNFs scaffolds (Figure 7 D and E). In contrast to TCP on day 1, neither CNFs scaffold stimulated Saos-2 cells to produce IL-Ra. However, by day 3, CM-CNF promoted the production of IL-Ra, IL-10, and IL-13, whereas TO-CNF failed to induce IL-Ra production. Interpreting individual cytokine production can be challenging and specific cytokines were therefore grouped as pro- or anti-inflammatory for analysis (Figure 7 C and F). On both day 1 and day 3, both CNFs scaffolds induced significantly lower total production of pro-inflammatory mediators from Saos-2 cells than from TCP. Conversely, TCP induced significantly higher total anti-inflammatory mediator production from Saos-2 cells than from either CNFs scaffold. TO-CNF exhibited significantly lower total levels of anti-inflammatory mediators than CM-CNF.

In contrast to Saos-2 cells, PDL fibroblasts secreted MCP-1 and IL-Ra on both day 1 and day 3 in all groups (Figure 8). On day 1, no significant intergroup differences were observed for any pro- and anti-inflammatory mediators (Figure 8 A and D). On day 3, however, significant intergroup differences were observed for all secreted mediators except IL-10 (Figure 8 B and E). Cells cultured on TO-CNF showed significantly reduced production of both pro- and anti-inflammatory mediators compared with those cultured on CM-CNF. With the exception of RANTES, MCP-1 and VEGF, there were no significant differences between the TCP and CM-CNF groups. Furthermore, TO-CNF stimulated PDLF to produce significantly lower levels of pro-inflammatory mediators than either TCP or CM-CNF groups. In addition, on day 3, the collective production of pro- or anti-inflammatory mediators by PDLF cells was significantly lower than in the TCP and CM-CNF groups (Figure 8 C and F). Taken together, these results indicate that the *in vitro* inflammatory response to CNFs scaffolds is mild and sometimes less than that of TCP. Furthermore, it is evident that the different cell types exhibit varying responses to the surface properties of CNFs materials.

### *InVivo* Inflammatory Response

**Figure 9:**
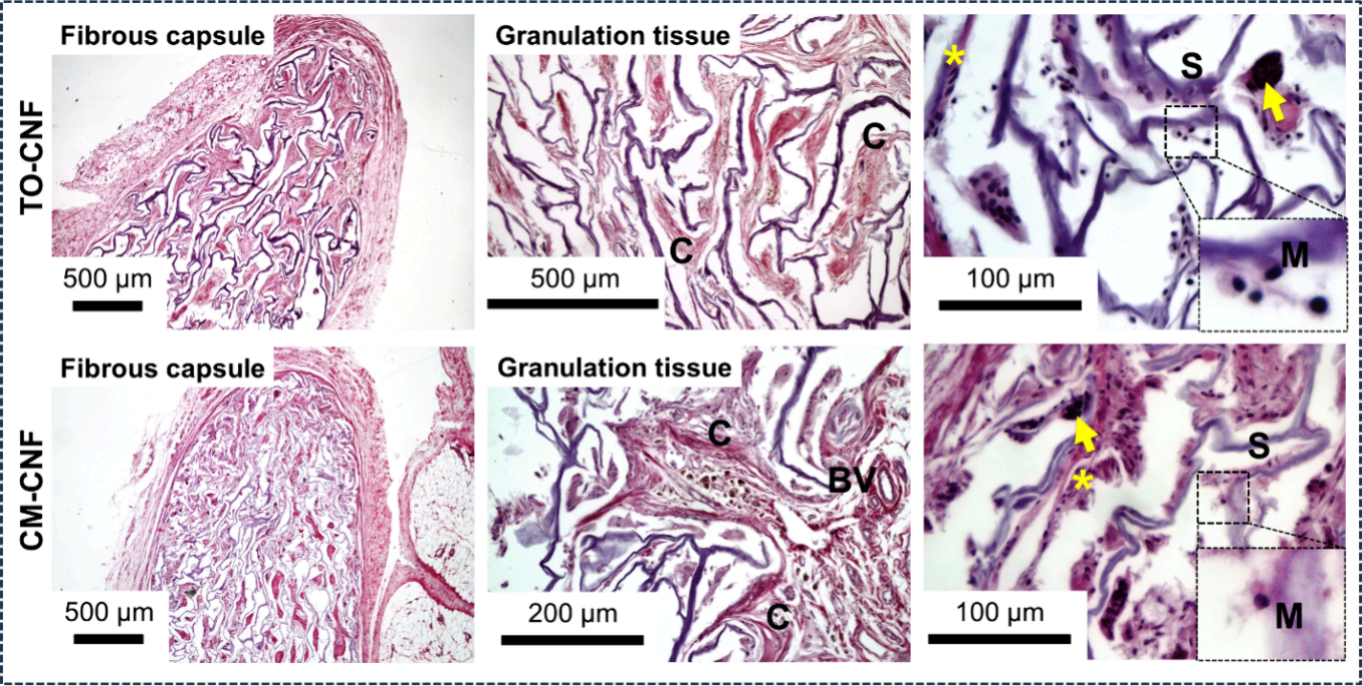
Representative H&E histological sections show the delayed host response to the implanted scaffolds, 90 days after subcutaneous implantation in rats. Both CNFs scaffolds induced a foreign body reaction (FBR). The scaffolds (S) were surrounded by a fibrous capsule and their pores were infiltrated with granulation tissue consisting of fibroblasts (asterisk), macrophages (M), foreign body giant cells (arrow), blood vessels (BV), and collagen matrix (C).

Figure 9 shows that both CNFs scaffolds were surrounded by an obvious fibrous capsule and their pores were filled with granulation tissue, comprising collagen matrix, fibroblasts, blood vessels, macrophages and foreign body giant cells (FBGC). Both scaffolds retained their original shape, with some weak signs of degradation, such as fragmentation and fibrillation of the walls.

## Discussion

The chemical modification of CNFs through TEMPO-mediated oxidation and carboxymethylation produced nanocellulose materials with varying chemical groups, as well as differences in charge, wettability, and fiber dimensions. While some studies suggest that specific surface characteristics can enhance protein adsorption on biomaterials, others report opposing results or no significant correlation. This discrepancy highlights the complexity of the issue, as protein adsorption and biological responses are influenced by multiple interacting factors. Importantly, it is challenging to attribute these responses to a single surface property, as the combined effects of various physicochemical factors likely play a more significant role in determining outcomes.

In addition to the complexity of biomaterial properties, the nature of the protein solution also significantly affects the results. Unlike simple protein solutions, which consist of a limited number of well-characterized proteins, the use of complex solutions offers a more comprehensive understanding of protein adsorption and its implications for material performance in biological systems. Fetal bovine serum has been identified as the protein adsorption model for our work because it offers several advantages. It closely mimics the protein composition of human blood plasma, is commercially available, widely used, and relatively consistent in its composition, making it a reliable and reproducible choice for studying biomaterials in physiological environments.^10,39^ Furthermore, all the cells studied with nanocellulose in the current research, as well as in our previously published work, were cultured in media containing FBS.^20,24^ Since these cell types depend on proteins adsorbed from the culture media for attachment and growth, using FBS is particularly relevant, as our protein model is crucial for replicating the environment in which the cells are cultured. This approach facilitated effective observation of their interactions with the proteins present in their culture environment.

### Effects of Surface Chemistry

TEMPO-mediated oxidation and carboxymethylation facilitated the breakdown of cellulose fibers into nanofibrils and microfibrils by introducing negative charges to the fiber surfaces, causing them to repel each other by electrostatic repulsion.^40^ Introducing different surface groups allows the production of nanofibers with varying charges, contact angles and sizes, as reported by other studies, and demonstrated in our current research.^28,29,33,34^ Moreover, the influence of surface chemical groups and their charge on protein adsorption, and consequently on cell adhesion, has been reported by several studies. Carboxyl-terminated surfaces have been shown to enhance cell adhesion by adsorbing cell adhesion proteins such as fibronectin and vitronectin, whereas hydroxyl groups, with their neutral charge and hydrophilic nature, result in poor protein adsorption.^9,11^ Moreover, aldehyde groups can promote cell attachment by reacting with the amines of adsorbed proteins via a Schiff base linkage.^41^ Additionally, McClary et al. suggested that carboxyl groups not only promoted high fibronectin adsorption but also encouraged the protein to adopt a favorable conformation, exposing cell-binding domains.^42^ Therefore, in the current study, the partial substitution of hydroxyl groups with carboxyl and partially aldehyde groups through TEMPO-mediated oxidation in TO-CNF samples led to an increase in cell adhesion, while modification with carboxymethyl groups did not improve cell adhesion.

We previously reported that the TO-CNF used in the current study has a higher carboxyl group concentration (COOH = 0.76 mmol/g) compared to CM-CNF (COOH = 0.058 mmol/g), due to the presence of carboxymethyl groups (CH_2_COOH = 0.34 mmol/g).^20^ In a noteworthy study by Hua et al., they demonstrated that human fibroblasts and Saos-2 cells showed reduced adhesion and a round cell morphology on unmodified nanocellulose compared to nanocellulose with carboxyl surface groups.^34^ They reported that carboxyl groups at > 0.26 mmol/g were required on the surface of TO-CNF materials to enhance cell adhesion and morphology. In another study, Hatakeyama et al. prepared TO-CNF materials with varying carboxyl content (0.31 to 1.60 mmol/g) alongside CM-CNF with 1.03 mmol/g carboxyl groups.^28^ Interestingly, low (below 0.59 mmol/g) and high (above 1.2 mmol/g) surface carboxyl group densities on TO-CNF negatively affected fibroblast proliferation and morphology. These conditions inhibited cell spreading and led to cell clustering, similar to our observations. To this end, it can be inferred that in our study, the presence of carboxymethyl groups and the low concentration of carboxyl groups in our CM-CNF samples are likely key factors impairing cell morphology. However, it has been reported that CM-CNF with 1.03 mmol/g of carboxyl groups also caused clusters of rounded fibroblasts with poor adhesion and morphology, suggesting the likely profound role of the carboxymethyl group.^28^ Understanding the impact of pretreatment methods on the molecular structure of nanofibers could help explain these seemingly contradictory results. It has been reported that carboxymethylation introduces carboxymethyl groups in a random manner, which is expected to disrupt the regular arrangement of carboxyl groups on the cellulose surface.^43^ In contrast, TO-CNF exhibits regularly repeating carboxyl groups on its surface, forming a characteristic alternating copolymer of β-1,4-linked D-glucopyranose and D-glucuronate.^44^ This regular alignment of carboxyl groups, similar to the structure of hyaluronan in the extracellular matrix, may play a significant role in enhancing cell attachment and proliferation.^28^

### Effects of Roughness, Wettability and Charges

In addition to surface chemistry, surface roughness of nanocellulose materials have been reported to influence protein adsorption, and consequently, cell attachment and morphology. When nanocellulose fibers aggregate to into a considerably larger surface fiber diameter (80 nm), they supported better cell morphology than nanocellulose with smaller fibers (20– 30 nm).^29^ The impact of polymeric nanofiber size on cell response has been shown to be linked to focal adhesion complexes in mediating cell adhesion. Nanofibers smaller than a critical size (1000 nm) were found to be ineffective in providing proper guidance cues to cells.^45^ This is due to the relatively large size of focal adhesion complexes compared to the fiber diameter.^45^ In agreement with that, the fiber size on the surface of TO-CNF samples in the current study was slightly larger (1100 nm) compared to CM-CNF surfaces (900 nm), suggesting more sites for cell attachment.

It is worth noting that protein adsorption on biomaterials is not determined solely by the surface chemistry and roughness. The chemical pretreatment of CNFs has been shown to result in alterations to its surface wettability and charges due to the introduction of different functional groups.^28,29,34^ Cells tend to adhere effectively to surfaces with moderate contact angles in the range of 40–80°. Arima et al. found that the number of adhered endothelial cells was highest on a mixed substrate with methyl and hydroxyl groups, and a water contact angle of 40°.^46^ Cell adhesion increased when the contact angle was between 60° and 70°, then plateaued on surfaces with methyl and carboxyl or methyl and amine groups. Our CM-CNF exhibited a higher contact angle (65° ± 1.1) compared to TO-CNF (35.3° ± 0.4), indicating better wettability for cell adhesion. However, CM-CNF did not support the elongated morphology of both Saos-2 cells and fibroblasts, suggesting that wettability may not be the primary factor influencing cell morphology in our scaffolds.

Since most proteins in the cell culture environment are charged, surface charge can have a significant impact on protein adsorption through electrostatic interactions between the surface and the protein. Our CM-CNF exhibited a zeta potential of – 29 ± 3.6 mV, while TO-CNF had a zeta potential of – 49.3 ± 4.7 mV. It was reported that the carboxymethylation process shifted the zeta potential of wood CNFs from – 7.5 mV to – 27 mV at pH 7, while condensation with glycidyltrimethylammonium chloride results in a positive surface charge (+26 mV).^29^ Similar to our results, both unmodified and carboxymethylated CNFs films were reported to promote poor cell adhesion and a round cell shape. On the other hand, cells cultured on the positively charged CNFs film adhered in large numbers and displayed an elongated fibroblast morphology. Positive charges may enhance cell attachment through electrostatic interactions with the negatively charged proteins. Interestingly, the authors observed the opposite trend with algae-based nanocellulose.^29^ Films with a positively charged surface did not provide favorable conditions for fibroblast growth and morphology, while negatively charged TEMPO-oxidized algae-based nanocellulose supported excellent cell morphology. FBS in the cell culture medium has both negatively and positively charged proteins which could be adsorbed onto positive and negative surfaces nonspecifically.^9^ The region of the protein which is in contact with the surface material upon adsorption does not necessarily bear the same charge sign as the overall protein. Moreover, the negatively charged regions of the protein may be shielded from the surface and its repulsive effect by the presence of counterions.^47^ These findings highlight the complexity of protein adsorption on charged nanofibers and the challenges of correlating a single surface feature with the biological response of nanocellulose.

It has been hypothesized that the surface properties of polymers can influence cell behavior either by directly affecting the cytoskeleton (e.g., integrin receptors) or indirectly through the alignment and unfolding of proteins. It has been reported that cell attachment on TO-CNF is an integrin-mediated process.^28^ Consistent with this, both fibroblasts and Saos-2 cells in our study produced vinculin. However, the distribution of vinculin and organization of cytoskeleton actin filaments was more prominent on TO-CNF than on CM-CNF substrates. Generally, efficient cell adhesion to a substrate requires adhesive proteins, such as fibronectin and vitronectin, which are found in FBS in cell culture media. Hatakeyama et al. demonstrated that the amount of 10% FBS proteins adsorbed onto TO-CNF substrates was significantly higher than that on unmodified CNFs (without carboxyl groups).^28^ However, they also reported that the adsorption of cell adhesion proteins, like fibronectin, was nearly the same across CNFs substrates with varying carboxyl densities. In line with this, Our TO-CNF samples (both 2D and 3D) adsorbed more proteins than CM-CNF and TCP groups, but there was no significant difference in the adsorption of adhesion and cytoskeleton-regulating proteins, such as prothrombin, fibronectin, vitronectin, adiponectin, and fibrinogen.

Overall, we speculate that, unlike CM-CNF, TO-CNF supports better cell adhesion and morphology due to its higher content of carboxyl groups, which enhance protein adsorption, promote favorable conformational changes in adsorbed proteins, and stimulate elevated levels of intracellular calcium. These factors together improve the accessibility of cell-binding domains to integrins, leading to improved cell adhesion and morphology.

### Effects of CNFs Shape

Although chemical pretreatment introduces different surface groups and alters the surface properties of CNFs, the bulk properties of 3D scaffolds, such as porosity, largely depend on the fabrication methods. In this study, CNFs hydrogels were frozen at – 20 °C before freeze-drying, allowing ice crystals to serve as porogens, resulting in scaffolds of similar size and volume for both TO-CNF and CM-CNF. In a previous study, we showed no significant differences in porosity, pore size, or pore size distribution between these scaffolds. The resulting structures exhibited porous architecture with pores diameters, ranging from 7 μm to over 590 μm.^27^ The difference between protein adsorption on 2D and 3D porous surfaces lies primarily in the surface area, the nature of interactions, and the accessibility of binding sites.^48,49^ In a flat, smooth and nonporous 2D surface (such as TCP), protein adsorption occurs only on the surface, providing a limited area for interaction. This can explain why TCP samples demonstrated the lowest number of adsorbed proteins (114) than CNFs samples. However, TCP supported excellent cell morphology. TCPs are typically made of polystyrene, which undergoes surface functionalization to introduce biologically relevant chemistry such as carbonyl and amine groups.^50^ The presence of carbonyl groups has been associated with improved protein conformation and cell adhesion.^51^ In addition, our proteomics data revealed 8 unique proteins adsorbed on the TCP surface and 20 unique proteins adsorbed on the CM-CNF 2D surface with the potential to interfere with cell attachment. Moreover, the shape of the substrate influenced not only the number of adsorbed proteins but also their types. For example, the 2D form of TO-CNF adsorbed 89 unique proteins, while the 3D porous TO-CNF scaffolds adsorbed 47 unique proteins.

On 3D porous scaffolds, the adsorption is not only limited to the surface but also occurs within the pores. This increases the available surface area for protein interactions, allowing more proteins to adsorb and potentially interact more deeply within the material. The porosity can create multiple binding sites in a three-dimensional space, offering a greater capacity for protein adsorption. Proteins adsorbed to 3D porous surfaces may undergo more varied conformational changes due to the complex geometry of the pores.^48,49^ The surface chemistry of TO-CNF (carboxyl and aldehyde groups) allowed for more adsorbed proteins than carboxymethyl functional groups on CM-CNF. However, 2D forms of CNFs materials supported more protein adsorption than their 3D counterparts. While 3D porous surfaces offer a greater volume and potentially larger total surface area, the 2D fibrous nanocellulose films can adsorb more proteins due to its high surface-to-volume ratio, higher density of functional groups, better protein accessibility, faster adsorption kinetics, and more favorable protein conformation on the surface.^49^

### Inflammatory Response and FBR

One of the key findings of this study is that the impaired cell morphology observed on CM-CNF scaffolds is independent of the cell type, whereas the inflammatory response varies depending on the cell type. For example, PDLF fibroblasts secreted MCP-1, IL-1Ra, and IL-13 on both day 1 and day 3 in all groups, whereas Saos-2 cells did not produce MCP-1 and secreted IL-1Ra and IL-13 only on day 3. At present, there are two perspectives regarding the surface-mediated effects on the host response to biomaterials and the *in vitro* production of inflammatory mediators.^52−54^ One viewpoint suggests that the surface chemistry of certain biomaterials can influence inflammatory cytokine production patterns, potentially affecting the foreign body reaction outcome.^54,55^ In case of PDFL, the production of inflammatory markers was significantly affected by the surface chemistry of the CNFs, aligning with other reports. In contrast, another perspective argues that for nondegradable biomaterials, surface chemistries have no influence on the fate of the foreign body reaction.^52,53^ In the case of Saos-2 cells, the different surface chemistries of CM-CNF and TO-CNF scaffolds had no significant impact on the production of individual inflammatory markers. Consistent with our finding, Schutte et al. found that polymeric materials with varying chemistries did not cause notable changes in the inflammatory marker profiles (TNF-α, MCP-1, IL-6, IL-8, VEGF, IL-1Ra, and IL-10) *in vitro* and *in vivo.*(52,53) Our findings suggest that the effect of different protein signatures on both CNFs is dependent on the cell type.

PDLF fibroblasts and Saos-2 cells were selected for the current study because they are commonly used to assess both cell adhesion and inflammatory responses induced by biomaterials, as well as for periodontal regeneration studies.^56−58^ Cellulose-based biomaterials hold promise for periodontal applications, particularly as membranes for guided tissue regeneration. In such scenarios, nanocelluloses come into direct contact with fibroblasts, osteoblasts, and macrophages, necessitating an evaluation of their interactions. In the current study, both cells secreted a range of cytokines known to regulate immune cells and impact the outcome of FBR. For example, IL-6 modulates TNFα production, which in turn enhances phagocytosis and chemokine secretion. IL-8 serves as a primary chemoattractant for leukocytes, while IL-12 enhances phagocytic activity and increases IFNγ production by T cells.^59^ IFNγ, either alone or in combination with TNFα, activates monocytes and macrophages, which are attracted to the implantation site by complement factors MCP-1, and RANTES.^60^ VEGF, a pro-wound healing mediator, promotes neovascularization and is associated with FBR formation.^61^ IL-1Ra counteracts pro-inflammatory signals, while IL-10 suppresses the production of pro-inflammatory cytokines by immune cells. Both IL-13 and IL-10 play critical roles in polarizing macrophages from a pro-inflammatory (M1) to a wound healing (M2) phenotype. The functions of these inflammatory mediators in FBR have been studied extensively and summarized in several reviews.^52,59,60^

It is known that implanted biomaterials can provoke an immune-mediated foreign body response, ultimately leading to the formation of a collagen capsule around the biomaterial.^15,17^ In some cases, this response can significantly impact the performance of the implanted biomaterial. Implant-associated fibrotic encapsulation can lead to the failure of various medical therapies and devices, including encapsulated cell therapies, cardiac scaffolds, biosensors, and nerve guides.^15,17^ This occurs due to impaired diffusion of nutrients, drugs, or analytes, as well as poor impedance and tissue distortion. Numerous studies have shown that the initial steps of the FBR involve nonspecific protein adsorption, desorption and conformational changes.^7,12,13,16^ Proteins such as complement fragments, fibronectin, and vitronectin bind to macrophages and regulate the formation of biomaterial-associated foreign body giant cells.^15,62−64^ The arginine-glycine-aspartate (RGD) sequence in fibronectin and vitronectin facilitates macrophage adhesion and promotes their fusion into foreign body giant cells.^60,62,63^ Negatively charged surfaces with carboxyl and hydroxyl groups, such as our CNFs, have been shown to activate the complement system, which is responsible for the clearance of foreign bodies and apoptotic cells.^64^ Among these complement proteins, C3, that was found on both CNFs scaffolds in our study, plays a central role in the activation of the immune response, including monocyte adhesion and FBR initiation.^12^ Similarly, in the current study, fibronectin, vitronectin and C3 were adsorbed onto the surfaces of both CNFs without significant differences, despite variations in surface chemistries and other properties. This may explain the comparable FBR outcomes observed after 90 days of implantation in rats.

Our histological findings align with several reports on cellulosic materials, which have demonstrated a similar chronic inflammation phase characterized by macrophages, foreign body giant cells, and fibrous encapsulation.^65,66^ When tunicate-derived cellulose nanofibrils (TNC) were implanted subcutaneously in rats and compared to expanded polytetrafluoroethylene (ePTFE) as a control, the results indicated no significant differences between TNC and ePTFE in terms of degradation and foreign body reaction over 90 days.^67^ In all groups, a distinct FBR was observed, characterized by a large presence of macrophages and giant cells. In contrast, when wood-based TO-CNF and CM-CNF were compared to degradable gelatin scaffolds in rats, the gelatin degraded after one month, whereas the CNFs did not.^27^ Although gelatin induced more acute inflammation after 4 days than CNFs, the foreign body reaction subsided due to the degradation of gelatin, while it continued to progress with CNFs. In general, both degradable and nondegradable materials elicit some degree of foreign body reaction.^27^ However, as degradation progresses, this reaction subsides in degradable materials, while it persists in nondegradable materials, with macrophages driving the response.

In conclusion, the findings of this study demonstrated that both CNFs scaffolds were nontoxic *in vitro* and *in vivo*, thus exhibiting a notable ability to facilitate repair, which renders them promising candidates for addressing impaired wound healing. Nanocelluloses have proven effective in improving wound care for burn injuries, often serving as temporary wound dressings until re-epithelialization is achieved.^68,69^ Nevertheless, the slow degradation of these scaffolds remains a challenge due to the absence of cellulose-degrading enzymes in humans and the tightly packed molecular structure of cellulose, which impedes hydrolysis. While these biomaterials are generally biocompatible, they can elicit a foreign body reaction due to their slow degradation, which is suboptimal for regenerative applications. To transition from a predominantly reparative role to a more regenerative one, modifications to the CNFs materials should be considered to mitigate the FBR and enhance their degradability.

## Conclusions

The chemical modification of CNFs through TEMPO-mediated oxidation and carboxymethylation produced nanocellulose materials with distinct chemical groups. Proteomic analysis revealed unique protein profiles influenced by the different chemistries and shapes of the CNFs materials. Numerous proteins associated with cell adhesion, cytoskeletal organization, and inflammation were identified on the CNF samples. Neither 2D nor 3D forms of CNFs materials, regardless of surface chemistry, exhibited cytotoxic effects on Saos-2 and PDLF cells. However, the introduction of carboxymethyl groups to CNFs surfaces led to impaired cell adhesion and morphology. We hypothesized that carboxymethylation of CNFs disrupts cell morphology due to their low density of carboxyl groups and the random incorporation of carboxymethyl groups on the nanocellulose surface. These modifications alter surface wettability, charge, and roughness, resulting in a distinct protein adsorption profile and potentially affecting protein conformation, though further investigation is required to confirm this. Despite differences in surface chemistry and proteomic profiles, both CNFs materials elicited the same foreign body response after 90 days of subcutaneous implantation in rats, indicating that the slow degradation of CNFs scaffolds is the primary driver of the foreign body response *in vivo*.
